# Barriers and facilitators to accessing support for people affected by rare dementias who are from culturally, ethnically and linguistically diverse backgrounds

**DOI:** 10.1186/s12939-025-02634-9

**Published:** 2025-10-09

**Authors:** Anna Volkmer, Jessica Jiang, Sebastian Crutch, Kerry Dathan, Emma Harding

**Affiliations:** 1 Division of Psychology and Language Sciences, UCL, London, UK; 2 Dementia Research Centre, Institute of Neurology, UCL, London, UK

## Abstract

**Background:**

It can take several years for people with rare dementias to receive a diagnosis. People from non-White and linguistically diverse backgrounds are also often diagnosed with dementia later than their White, English-speaking counter parts. These factors are likely to delay access to support for people who have rare dementias and who are from diverse backgrounds. This study aimed to investigate facilitators and barriers to people with rare dementia diagnoses who are from culturally, ethnically and linguistically diverse backgrounds in accessing appropriate diagnostic and post-diagnostic support services.

**Methods:**

Purposive sampling was used to recruit 10 people affected by a diagnosis of rare dementia who were from culturally, linguistically and/or ethnically diverse backgrounds. Semi-structured interviews explored experiences and perspectives in accessing care and post-diagnostic support. Reflexive thematic analysis was used to extract key themes.

**Results:**

Six themes were identified: (1) There is a lack of awareness amongst cultures perpetuated by intersectionality, (2) Carers experience tensions, (3) No society deals well with dementia, (4) Culture, language and ethnicity is a barrier in both directions, (5) Language as a barrier: languages spoken and language(s) lost, (6) What service providers need to do.

**Discussion:**

This study identified a lack of awareness of dementia within cultures as well as the wider community that was exacerbated by additional issues such as geographic, financial and gender disparities. Clinical care recommendations synthesised from the study results highlight a need to increase awareness of rare dementias within culturally diverse communities, as well as improving cultural competence within health and social care staff.

**Supplementary Information:**

The online version contains supplementary material available at 10.1186/s12939-025-02634-9.

## Background

It is estimated that up to 15% of the 50 million people living with dementia worldwide do not experience typical memory led Alzheimer’s or Vascular presentations [1]. These rarer dementias, often defined as non-memory-led [2] can initially affect skills such as personality, language and vision and are more likely to be younger in onset. These cognitive and associated non-cognitive difficulties, such as negative mental health or psychiatric difficulties, can often present unique challenges to people living with rare dementias, their families and the health and social care community [2]. Indeed, research has shown that it can take several years longer for people to receive a diagnosis of a rare than common dementia [3, 4]. Many never get the diagnosis they deserve, and underrepresented groups (including socioeconomically deprived groups) are more likely to only receive a generic dementia or dementia-unspecified diagnosis [5]. There is also evidence that people from non-White and linguistically diverse backgrounds are diagnosed with dementia later than their White, English-speaking counter parts [6–8]. This suggests that people from non-White and linguistically diverse backgrounds are more likely to have an even greater delay in getting a diagnosis of rare dementia.

A delay in diagnosis will inevitably delay access to treatments and support such as post-diagnostic support, speech and language therapy, counselling and psychological therapies, and pharmacological interventions [8]. Accessing services with a rare dementia is already challenging as many existing services have restrictive referral criteria (e.g., favouring stroke or typical Alzheimer’s [9]), may be delayed or unsuitable for people with rare dementia [10, 11] or simply less visible [2]. Yet the research on interventions such as pharmacological treatments and speech and language therapy for primary progressive aphasia, show that many of these treatments and supports are more likely to provide long-lasting benefits when started as early as possible [7, 12]. Importantly, delayed diagnosis has also been shown to lead to inappropriate use of services and excess spending on visits to accident and emergency and inpatient admissions [7].

People with rare dementias and their family members report difficulties navigating treatment and support [13, 14]. Inappropriate referrals and wasted time were felt by family members of people with rare dementia to restrict benefits of potential treatments [15]. Despite ethnic and linguistic diversity being identified as barriers to diagnosis in dementia, there has been little work to understand the intersection of dementia diversity and cultural, ethnic and linguistic diversity and whether these factors may pose additional barriers to accessing appropriate treatment and post-diagnostic support when living with rare dementia. To increase access to support, there is an urgent need to understand how these barriers are experienced and what could be done to facilitate improved access for people from these underserved groups and their families. This will inform tangible implementation strategies to facilitate greater engagement with tailored and appropriate support services for people from diverse backgrounds while also increasing the representation of culturally, ethnically and linguistically diverse people with rare dementia diagnoses in future intervention research studies.

With the ultimate goal to synthesise recommendations for health and social care professionals working with people with rare dementias to ensure people from culturally, linguistically and ethnically diverse communities can access their services, this study aims to investigate:

- What facilitated people with rare dementia diagnoses who are from culturally, ethnically and linguistically diverse backgrounds to access appropriate diagnostic and support services? (For the purposes of this study we have defined linguistically diverse backgrounds as those people who are non-native English speakers OR who do not speak English at home)
- What are the barriers to accessing these services for these groups and how these may be overcome?

## Design and methods

### Participants and recruitment

People with a diagnosis of a rare form of dementia and/or family carers who were from culturally, linguistically and/or ethnically diverse backgrounds (e.g. non-White British, non-native English speaking) were eligible to take part. Participants had to be over 18 years of age, able to participate in semi-structured interviews in English, residing in England/Wales and have capacity to consent to taking part in the study. Purposive sampling methods were used to ensure the perspectives and experiences of accessing care and support of participants from a range of diverse backgrounds were captured. Participants were recruited from Rare Dementia Support (RDS) (https://www.raredementiasupport.org/). This is a UCL-led collaborative service which provides support for people with seven different rare dementias including primary progressive aphasia (language-led dementias), posterior cortical atrophy (a vision-led dementia), behavioural variant frontotemporal dementia (which mostly affects personality and behaviour), Lewy body dementia (characterised by fluctuating cognition and motor disturbances), Young Onset Alzheimer’s disease (in which symptoms begin before the age of 65), and familial Alzheimer’s disease and familial frontotemporal dementia (both of which are inherited in an autosomal dominant manner) [16–22]. RDS membership data indicates that 92.75% of members identify as White, 3.19% identify as Asian, 1.86% identify as having a Mixed ethnic background, 1.54% identify as Black, 0.57% identify as Other and 0.09% identify as Arab. RDS members, whom RDS staff members identified as potentially eligible, were contacted via email and provided with an accessible information sheet about the study. Interested participants were invited to contact the research team to discuss the project further and to ask any questions. All participants provided informed consent to take part in the study, which included being video recorded. Interviews took place between April and June 2024. The study was conducted according to the guidelines of the Declaration of Helsinki and ethical approval for the study was granted by the Chairs of UCL Language and Cognition Department Ethics Approval Committee, Project ID: LCD-2024-01. (NB: Socioeconomic deprivation was not considered in the recruitment for this study)

### Data collection

This was a qualitative study using semi-structured interviews with people with rare dementias and/or their family carers. Semi-structured interviews are a useful method for exploring experiences and opinions and can easily be adapted to the needs of people with communication and cognitive difficulties, for example using images, key words or written information to support comprehension, and using prompting, and clarification to support expression [23]. Semi-structured interviews also allow for the emergence of unanticipated insights, and this was an important consideration given the diversity of experience and perspectives sought.

A member of the research team (KD) agreed a convenient time for the interview with participants either in person at the university or remotely via a video-conferencing platform (Zoom). Of the ten interviews, one was conducted in person at the university, at the preference of the participant. All interviews were video recorded.

Personal demographic data was collected including their age, diagnosis (or diagnosis of their family member), time since symptom onset/diagnosis, ethnicity and languages spoken. Semi-structured interviews were led by a researcher, KD, a speech and language therapist, trained in supporting people with communication needs. The interview topic guide included open questions exploring participants’ perspectives about how their cultural, ethnic or linguistic background might have influenced their experiences of accessing care and support related to a rare dementia diagnosis (see Appendices).

### Data analysis

All semi-structured interviews were video recorded, transcribed orthographically and reflexive thematic analysis was used to explore and create a coherent and complete account of the data. Reflexive thematic analysis is a useful strategy for identifying important ideas and is grounded in the data [23]. The data were analysed by two members of the research team (AV, EH). Initially, AV and EH familiarised themselves with five transcripts and generated initial codes which were then discussed, reviewed and refined into an agreed coding framework. This framework was then applied to all ten transcripts by AV and EH, supported by computer programs (Microsoft Word and NVIVO 14), and this further coding was reviewed at regular team meetings in order to generate a final set of themes which comprehensively represented the key patterns in the data in relation to the research questions. Collective analytic memos were written throughout the analytic process.

### Public involvement

The interview topic guide was informed by discussions arising from a Patient and Public Involvement (PPI) taskforce group established by the authors as part of a European-wide initiative (the INTERDEM Inequalities in dementia care taskforce; https://interdem.org/?page_id=7329) to explore barriers and facilitators to dementia care access internationally. Examples of work undertaken by the INTERDEM taskforce include exploration of inequalities in dementia care across Europe [24] and evidence on misdiagnosis of dementia [7]. A family carer of someone from a culturally diverse background who was affected by a rare form of dementia was invited to review the interview topic guide and study documentation for the current study prior to the interviews. Their comments and suggestions were incorporated to improve the accessibility and inclusivity of the study documents and procedures.

## Results

Nine recruited participants were carers of people affected by rare dementia, one participant – the partner of a person – attended with the person with dementia. Table 1. provides demographic information about participants in the study.

**Table 1:** Participants demographic details

Participant ID	Diagnosis of person with dementia	Relationship to person with dementia	Self-reported symptom timeline	Ethnicity/linguistic background of PwD	Ethnicity/linguistic background of carer
1	PCA	Husband	8 years	French; French speaker	White New Zealander; English and French
2.	svPPA	Wife	15 years	Indian; Gujrati/English/Hindi speaker	British Indian; Gujrati/English
3.	bvFTD	Husband	6 years	Hong Kong; Cantonese/Mandarin	Hong Kong; Cantonese/Mandarin
4.	lvPPA	Daughter*	6 years	Black British-Caribbean; English	Black British-Caribbean; English
5.	YOAD	Daughter	10 years	Black Caribbean; English & French	Daughter described herself as half British half Caribbean; English
6	bvFTD	Daughter (bereaved)	13 years, died 2022	Bangladeshi; Bengali and English	Bangladeshi; Bengali and English
7	PPA	Wife (bereaved)	8 years, died 2021	British Indian; English	British Indian; English
8	svPPA	Wife	7 years	British Asian; Punjabi and English	British Asian; Punjabi and English
9	lvPPA	Daughter*	6 years	Black Caribbean; English	Black Caribbean; English
10	lvPPA^	PwD (wife) + Husband	4 years	Black British; English speaking	White British; English speaking

*PwD* Person with Dementia, *PPA* Primary Progressive Aphasia, *svPPA* Semantic variant PPA, *lvPPA* logopenic variant PPA, *PCA* Posterior Cortical Atrophy, *YOAD* Young Onset Alzheimer’s Disease, *bvFTD* Behavioural variant Frontotemporal Dementia

*These participants were sisters, who wanted to participate separately

^This was the only dyadic interview with the PwD and their partner

### Thematic Analysis

The themes reflect the recurring important and understood ideas for the study participants and whilst bounded and agreed to be separate, do have interactions with the other themes. This speaks to the complexity of the core themes and the fact that this study’s results describe experiences that traverse both the experiences of participants affected by a rare condition, a progressive disease that changes over time, and in their identities as intergenerational family members from culturally and linguistic diverse backgrounds. Six main themes were identified in the data; (1) A lack of awareness is perpetuated by the intersection between dementia, rare dementia and aspects of our cultures; (2) Carers experience tensions in their roles caring for family members with rare dementias; (3) No society deals well with dementia, rare dementia is even worse; (4) Culture, language and ethnicity is a barrier in both directions when living with rare dementias; (5) Language as a specific barrier in rare dementias: languages spoken and language(s) lost; (6) What service providers need to do to support people affected by rare dementia. There is no hierarchy to these themes, and each was identified as equally important. The following provides a more detailed description of each theme.

Theme 1. A lack of awareness is perpetuated by the intersection between dementia, rare dementia and aspects of our cultures.

This theme describes how participants experienced a lack of awareness of dementia and rare dementias, and how the diagnosis is, or was, dealt with within their individual cultural communities. Participants reported a lack of awareness of dementia in general, which was amplified for rare dementias, amongst their cultures including Caribbean and Indian cultures. English words for dementia were often technical and the words associated with dementia in some other languages were felt to amplify the lack of understanding and consequent stigma associated with the disease. In fact, the word that is most commonly used to describe dementia in Bengali relates to ‘madness’. These attitudes seemed to exacerbate and be perpetuated by a lack of awareness of dementia and particularly rarer forms of dementia, creating a self-fulfilling cycle in which rare-dementia care needs were not fully understood and leading to a lack of engagement with and provision of services which could meet them.

“I truly believe that there is a cultural bias in the Asian community to not want to address anything related to dementia or Alzheimer’s, because we don’t have a word for it. There’s no word for dementia, by the way, in our culture… I don’t know 100% for sure, but I dare say India doesn’t have a word for dementia. You know. I think anything where someone’s acting a bit strange would be viewed as a spiritual madness sadly. And that is why people don’t want to talk about it. They? We have a very horrible word in Bengalis called “পাগল” (“bugol”), which means mad. So people. So this is how somebody would describe you. They would say, you’re mad they wouldn’t say, Oh, we don’t think that person’s well, they just say, Oh, “পাগল” was such a horrible word! I can’t, you know. Mad doesn’t really translate very well. It’s almost like saying psycho in English without using the word psycho. [P6]

Participants reported that within their communities even the most common forms of dementia were rarely talked about. This was attributed to a wish to not address the issue of dementia, and when it was discussed “it falls on deaf ears” [P6], meaning people did not believe in it. Consequently, people were unlikely to seek a diagnosis, let alone pursue a rare diagnosis, or ask for care and support for this stigmatising disease:

“If we don’t have a word for dementia, if the community is scared by the disease and thinks of it as a spiritual madness, who is going to seek help?” [P6].

Age was felt to exacerbate the lack of awareness of dementia within cultures and communities. Often participants described how older family members and community groups were unable to access up to date information because they were unable to use information sharing systems such as the internet. That information about rarer, often younger onset forms of dementia is less accessible and available generally, exacerbated this issue. Information about dementia was not tailored to relevant communities meaning that beliefs, specifically shame, were difficult to shift. Participants felt that respected institutions within communities such as spiritual centres did not assist in promoting any change in attitude, often exacerbating these feelings of shame. The lack of awareness of dementia combined with the shame imbued on the diagnosis of dementia for people from within families and cultures influenced whether people sought access to any kind of support, let alone tailored support for rare dementia:

“In Hong Kong there would be shame for my family” [P3]

“I think there is a problem (with) the cultural shame and my mum and dad didn’t want to admit my dad had dementia for ages, ages and ages and ages….I do think there’s a big shame about mental about being outside the norm” [P7].

Participants explained that when dementia overlapped with other barriers and other minoritised or oppressed characteristics such as having younger onset dementias, and being female and Black, this made things even more difficult. Participants reported a lack awareness of young onset dementias and their atypical features and experienced additional impacts of navigating a dementia diagnosis while still of working age. Cultural attitudes towards caring varied among the participants and were particularly incompatible with the structure of the care system and common beliefs about the responsibilities of care here in the UK. Specifically, carers described dominant beliefs and expectations that care should be provided by the family, rather than by an external organisation or services. These beliefs were often accompanied by fear of judgement from their community if they were to access care or support externally, as well as concerns that the person living with rare dementia’s preferences would not be met. For some this appeared to be underpinned by a sense of responsibility to, and respect for, the elders within their community and the value placed on intergenerational family life. For others there was an alternative or additional felt sense that it was in some way wrong to claim support or help and there was a sense of pride to be had in managing alone and not being reliant on wider systems for support.

“You think you can cope, you should be able to look after your husband, and you should be able to look after things yourself, and that is another… if it’s an older person thing or an Asian thing. But my mum feels that she should be able to look after a husband… She made that promise, as well to sickness and health… and we keep saying… there’s nothing wrong with getting help to keep that promise”. [P7]

“Yeah, because I think Asian community would think “Well, it’s our problem, isn’t it We’ve got to sort it out ourselves.” [P2]

“it’s all about family - family should look after family, not other people.” [P7]. That support services and systems specifically tailored for those with rarer dementia diagnoses and needs are sparse only served to perpetuate this as a barrier.

Equally, men with dementia from minority groups were reported as belonging to subcultures that made it harder to address or acknowledge the rare dementia symptoms. Finding support that was tailored to a given person’s specific combination of characteristics was also challenging. For example, if there was a culturally relevant care or support option it was often not age appropriate, or rare dementia-specific, or geographically accessible.

“Where I do struggle is finding, I think because she’s early onset,… she always feels much younger than the rooms that she walks into. So, she’s still very- even though she’s got bad hips, she’s still like very lively and she- and you go into these rooms and often it’s like lots of people sort of standing around or sitting sort of silently and there’s not a lot of life to it. So I think that’s definitely an issue. And she still wants to, she’s kind of stuck between, she wants to go back to doing the things that she used to do. But if we try and do that it becomes quite stressful for her and she can’t do it. She’s not ready to like sit in a room and play Bingo. So I think there’s a gap there.

The second thing I’d say is probably like culturally it’s also a bit of a misfit. I think often, even when people are talking about music, they’re kind of playing like songs from the Second- [inferred: World War]” [P5].

Similarly, poverty was often felt to exacerbate issues, and organisations that do provide information about rare dementias were considered somewhat exclusive in that the locations for support were hard to access for people with less money or work commitments, or for those living in geographical locations that were particularly rural or a great distance from the major cities in which tailored support and activities were being provided. Funding was identified as an issue beyond the individual level too, in that where there were examples of culturally appropriate rare dementia care and support, these were often facilitated by volunteers and lacked the long-term funding, resource and infrastructure to enable continuity of this care, something which was of heightened importance to those who had struggled to find relevant care and support or to feel a sense of belonging within these services:

“Where is the funding? Where is the money? So for some people it’s probably a thing where it’s like, what’s the point? Even me trying to access, because the rate now we help money funding resources out there for us is not what’s the point. I don’t have time. I’ve got 10 kids. I’ve got like a whole family to feed. I’ve got two jobs I’m trying to do here, like Mum or Dad or auntie, whoever it is. It’s just like, you know they got needs. And we’re ready holding on.” [P4].

Cultural differences in attitudes and perceptions in relation to rare dementia diagnosis processes and care were also evident in the way healthcare and support systems were organised cross-culturally. This was seen to trickle down into the macro levels of service provision as well as the micro level of individual interactions with services. The hierarchies of power and dynamics within medical appointments could act as barriers to the person living with rare dementias and their families being able to challenge a misdiagnosis or pursue a more specific rare dementia diagnosis e.g.:

“One of the things that I’ve realized is that in the French medical system, and it is a good medical system, but I think doctors and anybody in the system are sort of … or like to be considered as demigods. So, what they say is that. It’s not sort of how do you feel about this? What do you feel about that? It’s just delivering the message. And that’s it. There’s no sort of discussion about it” [P1].

Theme 2. Carers experience tensions in their roles caring for family members with rare dementias.

This theme highlights the tensions experienced by family members trying to care for the person with rare dementia in the context of different cultural communities. Family members found it difficult to negotiate these cultural barriers. Several carers described tensions being raised as western children to Asian parents, torn between their culture and their privileges as educated people who had developed skills that allowed them access to a vast range of information, so they understood dementia and could access the harder-to-reach information about rare dementias. The carers interviewed in the present study often took on a wealth of roles to attempt to access the relevant tailored care and support for their family member with rare dementia – these many and varied roles included being an advocate, an interpreter, a researcher and a care provider themselves, e.g.:

“I was always there to translate if it needed translating, or to interpret or communicate” [P1].

“…there must be loads and loads of people like my husband on the increase. And they may not have a lovely family like my husband has. What’s happening to them? [tearful] So. Yeah, it’s been hard. Getting the right type of care is hard.” [P2].

Some participants felt the first generation had to be independent, and experienced more barriers than they did, while being the second generation privileged them in some way. Many also described holding different views and attitudes to their siblings and others they were trying to negotiate care with, acting as a further barrier. Other carers reported trying to disguise themselves against any potential racism by speaking on the phone or using emails so organisations wouldn’t discriminate:

“I didn’t want them to be excluding (name) because they thought he might be in a different ethnic minority.” [P7].

Participants experienced wider ongoing tensions, including between employers who did not understand rare dementia or the cultural needs to care for people with dementia. Attitudes towards older generations or people with rare dementias was felt to be different across different cultural groups. Western cultures were felt to lack respect for elderly people at times. This was often exemplified by care agencies not taking culture or gender into consideration or even in the way people communicated:

“In the Caribbean culture like it’s very, like you’re very respectful towards your elders and you wouldn’t really, kind of, yeah, I guess like tell them off or chivvy them or kind of be over-familiar. And I think, yeah, like I’ve seen her in scenarios where she kind of comes in and she’ll sit down somewhere and people will be like “Let me just move your chair in and put you here” [participant gestures doing this] And like that really grates with her because she’s obviously like an adult woman and she doesn’t want to have her chair like pushed in for her like a child.” [P5].

There were also felt to be several barriers in the approach to medicine, for example being unable to give herbal or alternative medicines to loved ones:

“Until she was admitted to the hospital. Then we were not allowed to give her anymore CBD oil. We are not allowed even to give her some vitamin supplement” [P3].

Importantly, these tensions were also felt within communities. Even when carers were able to identify ethnically or linguistically appropriate services, these services either did not consider the individuals personal culture needs or were unable to cater for the specific symptoms of rare dementia:

“They came with their own biases. When my mom was making those sounds, I remember one person saying, Oh, yeah, do you read the Quran to your mum? And I was like, No, oh…. This is people that work for a care agency. If my mum was really into the Quran. It might make her feel better, but I can’t say she was somebody that you know diligently read the Quran every day, so that gives you an example of the insight of people in the in the so-called care profession from my community.” [P6].

Theme 3. No society deals well with dementia, rare dementia is even worse.

Participants described barriers and facilitators in dementia care generally, for everyone, regardless of their cultural background. The intersection between rare dementias and language loss associated with many rare dementias was one example that created even bigger barriers. Whilst staff could occasionally accommodate speaking another language such as French, they were unable to deal with people with no retained language skills at all:

“Well, they didn’t know they didn’t know how to deal with it, because they couldn’t communicate with her, and they didn’t seem to find any way of communicating with her. So, you know they just did what they felt was correct rather than anything else, and then, if there was anything, they didn’t communicate with me if you like, and sort of say “could you please tell us what she could have here, or what she needs here, or whatever” [P1].

A general lack of awareness of dementia was felt to span cultures, crossing beyond ethnicity and culture. People simply do not understand and often fear dementia, regardless of ethnicity or culture. Rare dementias were considered even worse and described as violent and lonely, and out of control giving a sense of the isolation that people experienced regardless of other barriers associated with ethnic or linguistic issues. Building on this, one participant described the specific barriers in the context of rare dementia when they explained that their communities are not considered hard to reach in relation to access to support and awareness for other conditions such as diabetes or heart health:

“We’re often described as hard to reach communities. Right? It’s a very negative way of describing our communities. And you know, like I go back to diabetes. But we’re not hard to reach when it comes to diabetes right? We’re not hard to reach when it comes to weight management. So why are we suddenly hard to reach right if you have services that are all hidden away?” P6.

The lack of awareness within cultural communities highlighted something specific about dementias – and exacerbated or magnified in the case of rarer, lesser-known forms of dementia – that cause these populations to face specific barriers to accessing care and support which is attuned to and appropriate for them. Participants emphasised that dementia care needs to change more broadly, and expressed concerns that if their experiences are in anyway similar to how everyone is treated, then there was a much bigger issue at stake:

“It sad state of affairs. But we can only hope that everybody is experienced in the same level of service and care. But if they are like, I said, it’s still woeful because if this is, if our experience is indicative of other people’s experiences. Thenwe are in a heap of shit” P4.

Participants advocated for dementia care than spans cultures and focuses on quality over quantity of life. They highlighted that care needs to cater for all cultures because culture *is* quality of life. Related to this, several participants articulated the potential and perceived significant impacts on their family members affected by rarer dementias because of these gaps in culturally sensitive and appropriate care. This was conveyed as an experience that could be detrimental to a person’s inherent sense of personhood and identity and that could diminish their ability to engage with the world around them, connect to others, feel a sense of belonging and participate in the activities of everyday life:

“Cognition is only one part of a human experience, you know… They’ve still got other abilities. But cognition somehow seems to get such a bad rap that you’re nobody. You’re no longer cool, because your conversation doesn’t make sense.” (P2).

“…finding care. I feel like I’m looking for something very specific. And I think I would, in an ideal world… Because… her memory will get worse and maybe she won’t recognise this person or-. I think having someone who feels like culturally familiar is helpful, especially in your home, I think. So in my ideal world I’d love someone who’s like from a Caribbean background, like, probably quite young, probably would understands like what kind of music she wants to listen to, like, will dance with her, will do all the things I think make her happy… I know that I’ll probably have to try a few carers before we find the right person, but I think I’m just very nervous of finding a good fit… someone who brings like a bit of joy into the house, and is like light-hearted, and will dance… I just want her to have like a nice life as well as to be safe”. (P5)

Carers described their hopes for personalised rare dementia support which would keep their family members not just safe but stimulated, engaged, appreciated and valued, with a sensitivity to culture an important factor in enabling that.

Theme 4. Culture, language and ethnicity is a barrier in both directions when living with rare dementias.

Several participants expressed their own or their family member’s mistrust of healthcare professionals or systems as a barrier to their engagement with care and support pertaining to rare dementia. Participants cited previous experiences, perceptions or anticipation of institutional racism, prejudice or biases as being responsible for this:

“I think there’s definitely a thing where she feels more comfortable dealing with like doctors or carers who are Black or Caribbean, I think. It’s just because I think she thinks “at least I know”, like, “racism won’t come into play here”. (P5)

“I think she doesn’t trust doctors because she’s concerned about racism… I know she had a certain experience but I don’t know the details of it… but I know that probably in addition to I think other people’s experiences within our family, I think that probably also kind of means that she kind of does mistrust doctors.” (P5).

Prejudice within the health and social care system was sometimes constructed by professionals from within their own cultural communities. Not only did some people describe assumptions made about spiritual preferences and needs of people with dementia but this also resulted in inappropriate care, such as reading the Quran to people who would not ordinarily read it. Participants also felt that health care complaints or concerns were not valued or respected by people from within their own cultural communities. This inhibited the desire to pursue a diagnosis of rare dementias or access to tailored care and support. In one example, Indian health care professionals were described as treating people from Indian communities less seriously:

“I think Indians don’t take Indian seriously…I’m making a broad statement here. But I think, yeah, they just think like, Oh, you know, they have a different standard. So, my friend, who’s in the NHS, she said that as well she goes “We probably should not go to Indian doctor”… So I think that she said that and I went a couple of times, and I’m quite articulate. But he did fob me off. And I said, “Look, it’s quite serious. He doesn’t seem to be performing normally” and it’s only when I actually said, “No, I think I know my rights, and I need to, I want a referral via BUPA ‘cos I’ve got that, I’m paying for it so I’d like a referral letter, and let’s let them do the tests.” …So I think, yeah, I think it does make a difference. If you’re from an ethnic background, you’re not taken seriously. Yeah, you have to kind of say, you kind of… you have to be saying you’re exaggerated before they take you seriously. (P2)

Participants expressed concerns that some health and social care staff from diverse cultural groups had less understanding about the cognitive, behavioural and other specific symptoms associated with rare dementias. Despite families trying to explain and advocate for their loved ones, this was not always instrumental is assisting health and social care professionals understanding of rare dementia:

“Most of the staff dealing with [name] English was not their first language. Spanish was. I think there was a Russian and I think [name] found it really hard to understand them.…He was in a geriatric. He was in a geriatric ward when he was in hospital. No one ever paid attention to what I told them… But actually, in the end I made two laminated sheets and I said, can you please put these on the head next to [name’s] notes and it basically says, you know [name] cannot read. He cannot write. He cannot use one of two, two or three times this” P7.

Theme 5. Language as a specific barrier in rare dementias: languages spoken and language(s) lost.

This theme captures the importance of language and the manifold ways it could be a barrier to the engagement with and access to care and support for those with rarer dementias. Being from culturally and linguistically diverse backgrounds, English was often not the first language of the people living with rare dementia described in this study, and many were no longer able to speak English due to a progressive language loss associated with their rare dementias. This meant it could be difficult (or impossible) for people living with rare dementia to understand those providing care and support or to make themselves understood.

“…she went into a hospital locally. And of course, the first of all, the staff couldn’t cope with somebody speaking just in French, so they just sort of made it up as they went along… Well they didn’t know they didn’t know how to deal with it, because they couldn’t communicate with her, and they didn’t seem to find any way of communicating with her. So, you know they just did what they felt was correct rather than anything else, and then, if there was anything, they didn’t communicate with me if you like, and sort of say “could you please tell us what she could have here, or what she needs here, or whatever”. And, you know, just to sort of have that involvement where, if they weren’t sure to ask a question rather than just sort of ignoring the problem effectively… [so they stopped asking] what she needed. If she needed toilet, if she wanted this or whatever. So she was just basically left to lie there, and when it was her turn to be attended to, they attended to that sort of thing.” (P1).

“I found there’s nothing out there because my husband did not speak English, so he used to go to Alzheimer’s a day centre, and he will sing all these songs, you know “It’s a long way to Tipperary”, you know, “You are my sunshine”. These songs don’t mean anything to my husband, because culturally, he can’t relate to them, and so he wouldn’t want-. I wish there was something Asian facility, so he could actually relate to the songs or the people, or he could feel part of it. So he was going there was pretty-. It was all White. ‘Cos that’s where I live in, is, you know, I live in [place name] and so there wasn’t anything there. They were lovely. They’re very kind, lovely people, so I’m not knocking them. They’re beautiful people. I really like them, and I was very happy. My husband was going there, it felt safe. But the challenge was, there was no language. You know there was nothing that he could gra-. He was already struggling, and then the language was, the English language has gone. ‘Cos you know this is FTD. The second language disappears. So you’re now left with your first language, and be living in a land which is English, and you can’t relate to it. So that was another challenge for him.” (P2).

Many of the people living with rare dementia being described in this study had experienced language-related symptoms due to their rare form of dementia, so for many it was not only that they didn’t speak the dominant language, but that their use of even their first language could be impacted in ways in which many health and social care professionals were unaware of or inexperienced with. Several carers had accessed speech and language therapy support for these progressive speech and language difficulties associated with rare dementias, and many had come up with their own systems for helping their family member to express themselves, but these were often not taken up or implemented in other care settings for example during hospital admissions:

“in terms of facilities as a baseline, you want a place where there’s compassion and there’s care and there’s love, so that can serve for all communities. You know irrespective of language, anyway. That’s the base point everyone should have. But then for people with ethnic and language challenges, you do need to consider that, you know, if they have dementia, that the second language is gone. And I remember one incident… they said, “Oh, actually, he can’t come here any longer”. I said “Why?” … And they said… “Well…other attendees are not happy with him…because he keeps on making that noise. Hmm-hmm-hmm”. ‘Cos my husband kept trying to communicate. Hmm-hmm-hmm. That was his kind of thing. So I said, “But you know that’s his condition.” I’ve come here because you specialize in dementia. Now, if a dementia centre says to me his dementia is too much of a dementia for other dementia patient, where do I go?”(P2).

Language posed multiple issues and for carers for whom English wasn’t their first language, it could be difficult to understand lengthy documentation and complicated terminology used in appointments. When they were offered access to support group meetings tailored to rare dementias the local and colloquial cultural references used made things more difficult to understand, e.g.:

“When British people are talking with each other, they use a lot of local culture, and, you know, the British people like to have small talk making a joke and everybody ha ha ha ha. That’s something I could not understand 90% of it. So I don’t go to the meetings with all the British people, not because I hate them. They are very kind. They are good to me. But I just can have difficulties to trace to what they’re saying.” (P3).

The impacts of this lack of understanding was described as significant, it could prevent carers from making connections, feeling a sense of belonging, but also understanding their family member’s rare dementia as well as treatment plans and their rights and entitlements. Experiences with translated materials and interpreting services were often limited as these were reported as almost never being available in relation to rare dementia.

Theme 6. What service providers need to do to support people affected by rare dementia.

This theme captures the many and varied suggestions and recommendations participants shared informed by their own experiences, that would improve access and quality of care for those affected by rarer dementias from culturally, ethnically and/or linguistically diverse backgrounds. Diagnostic tests and other assessment materials were reported by some participants to not be culturally sensitive or appropriate.

“If I go back to the memory test, right? That was done. It is communicated in English, right? Why is it only communicated in English?” P6.

There was a widely acknowledged need for culturally informed and attuned provision – notably this included linguistic support such as interpretation and translation but also included an expressed need for support which encapsulated culture beyond language, for example with relevant and familiar music, food and values being given consideration alongside rare dementia.

“They don’t serve the food that she like. They don’t listen to the music that she like. They don’t do the activities that she likes. They don’t watch the you know programs that she likes, and when she goes there she is the minority. So it’s kind of like what the majority loves” PT9.

Crucially, participants acknowledged that along with improved provision of culturally sensitive care and support for rare dementias, raising awareness of and encouraging engagement with this was a separate issue of equal importance. Participants suggested that making the most of existing relationships with relevant community groups and organisations (e.g. cultural and religious groups) would be a key method through which awareness raising and openness to engagement in rare dementia care and support could be fostered, building on existing trusted relationships, familiarity and a sense of comfort, representation and belonging – all things which were described as missing from some existing culturally misaligned care and support options:

“So it was a huge South London Bengali Association. They went on holidays together. Strong, strong community, very strong community. So when it comes to. I would say, any barriers where the issues might be cultural you know. Maybe what needs to be done is that the community leaders, leaders from the mosque leaders, from, you know, respected community members should be educating their groups on what dementia is.” P6.

While language was not the only factor in services and resources being made more accessible and inclusive to those from diverse backgrounds, it was a key one, and participants called for more readily available interpreter services for appointments, improved availability of translations of key documentation on rare dementia, flexibility in the format of appointments and materials (e.g. online versus in-person) and training for staff in accessible communication to improve understanding and ability to process what could be complex information at an often emotionally challenging time:

“I mean lack of accessible services. It’s a difficult one for me to answer, because I was always there to translate if it needed translating, or to interpret or communicate, or whatever. And, yeah, I mean that’s- I can’t really comment on that. Postcode lottery, definitely.” P1

“so training of staff in hospitals that they need to speak to people slowly, clearly, and in simple language that they understand.” P7.

One participant had themselves been involved in outreach work within their local community groups sharing their experience of supporting someone with a rarer form of dementia with those from a similar cultural and ethnic background to them. They had found this a rewarding and productive activity and called for the facilitation of further similar opportunities for those from other diverse backgrounds to be supported in outreach work within their respective communities to help break down barriers to access for people with rare dementias from their own communities.

“I have done some talks. We did one talk with the Bangladeshi community.” [P2].

Finally, capturing the way these culturally specific experiences give rise to specific support needs, one participant, in exploring their own attitudes to providing care for their family member with rare dementia, proposed that there may follow a linked need for specific support for people around the person who themselves will have unaddressed needs for care and support:

“the doctors and neurologists are focused on the patient, but they don’t sort of necessarily look at what could be done to support the-, not only the carers, I guess, but the people around. You know, our son and our daughter. I know that they, they’re really hurting with what’s happening to their mother. But there’s nobody there to sort of apart from me to sort of put an arm around them, and sort of look after them, or sort of do things like that, and-. There is no sort of blanket type of, no comfort blanket, I suppose, is the best way of putting it.” P1.

## Discussion

To our knowledge, this study is the first of its kind to explore the barriers and facilitators that people from culturally, linguistically and ethnically diverse backgrounds with diagnoses of rarer forms of dementia experience in accessing care and support in the UK. This exploratory study has highlighted both a broad lack of available and accessible culturally attuned care and support provision. Where culturally or diagnosis-appropriate care and support options do exist, there are further and extensive barriers to access. These are associated with practical factors such as funding and geographical location. Inter- and intrapersonal barriers were also identified, such as variations in cultural beliefs and attitudes to both dementia as a health condition as well as to caring responsibilities and care options. The experience of having a rare dementia and/or seeking support were associated with a sense of shame and stigma for many participants, resulting in substantial barriers for families from diverse backgrounds in engaging with tailored care and support for their family member affected by a rarer form of dementia.

The findings highlight how the layering of (i) minoritised cultural, linguistic and ethnic characteristics with (ii) disability, in the form of lesser-known types of dementia, works to exacerbate barriers to appropriate care and support for these underserved populations. This is aligned with and builds upon existing literature which highlights the way the intersectionality of certain characteristics (e.g. gender, race) can disproportionately exacerbate negative outcomes such as financial and emotional burdens experienced by dementia carers and their psychological and physical well-being (e.g. 25, 26).

Prior and current experiences of, and the anticipation or expectation of, systemic racism in healthcare and healthcare professionals posed barriers to access to both diagnosis and consequent care for those from diverse ethnic backgrounds. This is reflected in a recent systematic review which found ethnic background resulted in delayed and misdiagnosis across dementia types, but particularly Lewy Body Dementia and Frontotemporal dementias - both rarer dementias [7]. This review emphasised that people from diverse backgrounds were more likely to be attributed mental health diagnoses during the misdiagnosis process. This was similar to reports from participants in the current study, however and importantly, the current study highlighted that some of this discrimination was from health and social care professionals or carers from within their own cultural communities who attributed symptoms to anxiety or depression or were simply dismissive of reported symptoms. This aligned with some of the broader narratives within the cultures participants in the current study were reporting on, where dementia was ignored or shameful or attributed to ‘mental health’ rather than a physical neurological condition.

Current care services do not currently have the capacity to support people with rare dementia and people from culturally or linguistically diverse backgrounds. The current research evidence has demonstrated that services are unable to cater for people with rare dementias, leading to a postcode lottery and inequitable access to care across the UK [8, 27]. Cultural competence is a novel and yet under-implemented ethos, where guidance or recommendations is informally and therefore haphazardly applied [28, 29]. For the participants in the current study this meant that where it could be found, carers were identified as playing a significant role in sourcing care and support for their affected family members, often expending a lot of emotional energy and time to persevere in ensuring their family member received the support they required, often while navigating challenging dynamics with wider family and gaps, inconsistencies and inefficiencies in service provision. Language played a key role in the challenges faced in several ways – in the sense of the language skills and abilities that had been lost, particularly for those experiencing a form of primary progressive aphasia, along with the challenges associated with being non-native English speakers trying to way-find through complex services and pathways with minimal (if any) language-based support.

**Fig. 1:**
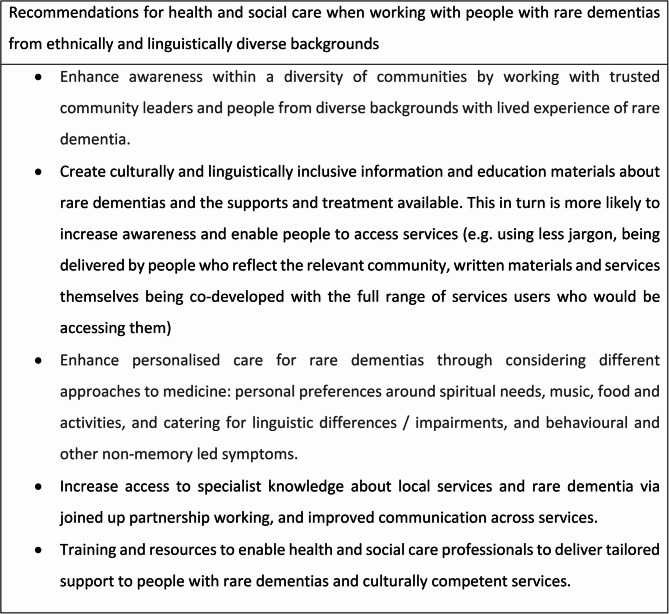
Synthesis of recommendations for health and social care when working with people with rare dementias from ethnically and linguistically diverse backgrounds

In having found their way to some form of rare dementia care and support and to this research study, participants were able to also share their experiences of what had facilitated their engagement with support and their suggestions for further improving access for those from similar backgrounds. These have been synthesised as a set of recommendations in Fig. 1.

While this study has focused on the experiences of people who have essentially found some support (i.e. in being aware of this research study), the researchers and participants themselves acknowledged that there are whole other communities of people whose experiences and perspectives have not been captured by this work, particularly those who don’t have any (or an appropriate, specific) diagnosis, and those who haven’t had any support at all. All the participants who identified as from culturally and linguistically diverse backgrounds in this study could speak sufficient English to participate, such that interpreters were not needed. It is possible that those who did not speak sufficient English might have identified different barriers to accessing support. Indeed, the purposive sampling strategy of people who *had* accessed support used in this study, perhaps indicates that people who do not speak English as a first language might have more acute difficulties in accessing support. Further outreach work involving community partners and facilitated by people with lived experience from diverse backgrounds would be valuable in engaging the yet unreached members of these underserved groups. The small sample size recruited to this study was considered to carry sufficient informational power [30], given the sample specificity was limited to those who had already accessed support. Despite this the participants are by no means homogenous in terms of diagnosis, linguistic or cultural backgrounds, therefore the recommendations outlined here are very broad and future research focusing on issues specific to each culture would be beneficial. Participants in the study referred to poverty as a factor that may exacerbate barriers to accessing support, yet this study did not actively recruit nor collect data on participants’ socioeconomic backgrounds. Future research on the current topic of barriers to accessing support for people affected by rare dementias should explore this issue further. However, participants emphasised the power of large research institutions to influence both within cultural groups and health and social care services. Importantly, as White British researchers, with one of our research team reflecting on their bias as the child of a migrant parent, future research should be undertaken with and by people from relevant cultural, ethnic and linguistic backgrounds. Given the language-based barriers identified in this study, further work with multilingual participants would be helpful in shedding light on how challenges related to this are experienced and can be best navigated and accommodated in future post-diagnostic support and care. In addition to this, the specific language-based challenges faced and culturally mediated emotional impacts highlighted in the current study (e.g. the experience of guilt in ‘outsourcing’ care) illuminate particular areas of potential within speech and language therapy and psychology intervention development that could be helpfully informed by research involving more diverse participant groups.

## Conclusion

This study describes the many layers of accessing a diagnosis and treatment or support for a rare dementia for families from linguistic, culturally and ethnically diverse backgrounds within an English and Welsh lens. A lack of awareness and presence of shame within their own cultures and communities as well as the wider community they live in was exacerbated by additional issues such as geographic, financial and gender disparities. Services that did exist were not able to not resourced to provide the persona centred support that they felt would be of most benefit to their family members with a diagnosis. Future research and clinical care must focus on how to increase awareness of rare dementias with culturally diverse communities, as well as improving cultural competence within health and social care staff.

## Supplementary Information

Supplementary Material 1.

## Data Availability

Access to anonymised data sets may be available by contacting the first Author.
