# Supplementary material for: Barriers and facilitators to accessing support for people affected by rare dementias who are from culturally, ethnically and linguistically diverse backgrounds
Source: Int J Equity Health. 2025 Oct 9;24:261. doi: 10.1186/s12939-025-02634-9 (PMC12512257; doi:10.1186/s12939-025-02634-9)
Supplement: Supplementary file 1 — Supplementary Material 1. [file 12939_2025_2634_MOESM1_ESM.docx]

**Appendices**

# **ERB barriers and facilitators for PLWRD from diverse backgrounds – topic guide for interviews**

**Introduction:**

**Get permission and** ***** Start recording *****

- **As you know, we are exploring the barriers and facilitators to accessing care and support for people living with rare dementias who are from linguistically or ethnically diverse backgrounds. You’re here because XXX has a diagnosis of a rare dementia. Thank you for joining me today to talk about your experiences and what you think could be done better.**
- **Do you have any questions about the information or consent form?**
- **Intro self: I’m Kerry, research assistant on the project run by Dr Anna Volkmer and Emma Harding, and I am SLT and researcher; worked with PW stroke and brain injury, but not with rare dementia.**
- **We will be about 60-90mins, if you need a break, or doorbell rings, let me know**
- **Recorded, might make occasional notes, remind me of things I want to ask you later**
- **Are you ready to get going?**

**Tips:**

- - Anything not clear: prompt to “explain”, “tell me more”, “example”
  - More detail: “what happened next?”, “how did that come about?”, “why?”
  - Validate what they’re saying is important
  - If ask for advice: will note down and ask Anna to get back to them
  - Keep on topic – keep coming back to the questions
  - Allow silences
  - Use their words
  - Note things to go back to

**Questions:**

**1. Can you tell me about your linguistic/ethnic/cultural background – how would you describe that?** [and your family member’s]

1. **Could you tell me a little bit about the type of dementia you/your family members/partner had/has?**
   - Probe their understanding of the diagnosis – e.g. What words or terms do you use to describe it?
   - How would explain your type of your dementia to a friend or neighbour?
2. **Can you tell me about how you got the diagnosis?**

- What barriers were there? (to getting the diagnosis)

- What helped you to get it

- Do you think your linguistic, cultural or ethnic background influenced this at all

**4. Can you tell me a bit about the types of dementia care and support you/your family member have accessed?**

Possible prompts:

Healthcare, social care, charities and other organisations, paid care, activity groups, social network (friend and family support)

**5. Do you think the languages you speak, your cultural or ethnic background have affected how easily you could access help for your dementia? (30 minutes: about here)**

Possible prompts:

- Specifically, **dementia care and support**?

-What helped you to get around that barrier (if you were able to)?

- Why was that not an issue for you? Tell me about that.

**6. People have suggested the following can be barriers to accessing care and support. Have they been for you (show or read list)?**

Lack of awareness – people not knowing about dementia

Lack of funding – for NHS and social care staff

Higher level systems e.g. policy, communication between professionals, wider politics, priorities (services designed for cure not care)

Lack of available services

Lack of accessible services (e.g. wrong / technical / visual / audio language)

Postcode lottery

Lack of personalised care/poor quality of care (e.g. cultures or symptoms)

Barriers to getting a diagnosis (e.g. time taken, misdiagnosis)

Stigma (societal, cultural)

Workforce (e.g. lack of skilled or culturally sensitive care, previous negative experiences)

Drug treatments/medication (e.g. lack of these, perceptions of Western medicine- people reporting taking alternative medicines)

Familial or community perceptions about accessing formal or ‘outside’ care and support

Denial

Shame

**7. What has helped you to access dementia care and support?**

Possible prompts:

Has anyone in particular helped you to access dementia care and support?

Have you found any types of support particularly accessible for you/your family member? What do you think it was that made them so?

**8. Do you have any thoughts about what might prevent others from a similar cultural or ethnic background to you from accessing dementia care and support?**

**9. How could dementia care and support be made more accessible for you/your family member and others from culturally and linguistically diverse backgrounds?**

**End:**

**Thank you very much for sharing your experience of accessing dementia care and support with me today. That’s the end of the questions. Is there anything you would like to add?**

**Thank you for your time today. Interviewing others. Analysing interviews well takes some time (months), but when we get to that point, we would happily share the results with you if interested?**
